# Intracranial Hemorrhage and Pneumocephaly After Cervical Epidural Injection

**DOI:** 10.5811/cpcem.2019.7.43859

**Published:** 2019-10-14

**Authors:** Nishit Mehta

**Affiliations:** Summa Health Barberton Hospital, Department of Emergency Medicine, Barberton, Ohio

## Abstract

Cervical epidural injections are commonly used to treat patients with radicular neck pain. The following is a description of a case of subarachnoid hemorrhage, subdural hemorrhage, and pneumocephaly following cervical epidural injection.

## INTRODUCTION

Cervical epidural injections (CEI) are frequently used to treat patients with radicular neck pain. There were 173,925 upper spinal epidural injections performed on Medicare beneficiaries in 2004.^1^ Common complications from the procedure are increased neck pain (6.7%) and headache (4.6%).^2^ The reported overall complication rate is 16.8%.^2^ However, the literature includes many cases of serious complications such as brain and spinal cord infarction.^3^ The pathophysiology of these complications is thought to be embolism of particulate steroid from accidental intra-arterial injection. The following describes a case of subdural hemorrhage (SDH), subarachnoid hemorrhage (SAH), and pneumocephaly following CEI.

## CASE REPORT

An 88-year-old female presented to the emergency department (ED) with headache following steroid CEI. She had the injection performed prior to arrival as an outpatient. She had received propofol 50 milligrams (mg) intravenous during her procedure. Following the CEI, she became bradycardic. She also reported headache, nausea, and vomiting. On presentation to the ED her headache was severe. She also reported left upper extremity weakness. Past medical history was significant for atrial fibrillation, lung cancer, chronic kidney disease, and hypertension. Her home medication was significant for aspirin 81 mg tablet daily with no other anticoagulant or antiplatelet agents.

On physical examination vital signs were temperature 36.2° Celsius, pulse 67 beats per minute, respiratory rate 18 per minute, pulse oximetry 98% on room air, and blood pressure 188/102 millimeters of mercury. On general exam she appeared distressed. Head was atraumatic. Her eyes had normal conjunctivae, normal extraocular movements, and her pupils were equal, round, and reactive to light. Neck was supple. Cardiovascular exam demonstrated a normal rate, regular rhythm, normal heart sounds and intact distal pulses. Pulmonary exam showed normal effort, with neither wheezes nor rales auscultated; however, she did have scattered rhonchi. Neurological exam showed her to be alert and oriented to person, place, and time. She followed commands appropriately, her gaze was normal, she had no visual field cuts, and no facial palsy.

**Image 1:**
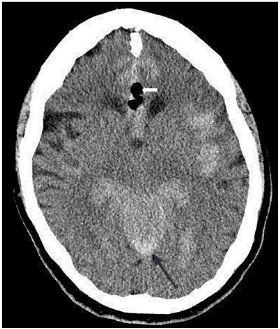
Computed tomography scan of the head showing pneumocephalus (white arrow), and subarachnoid hemorrhage (black arrow).

**Image 2:**
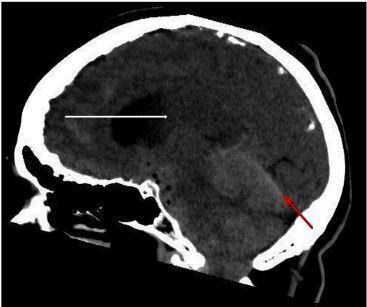
Computed tomography of head showing subdural hemorrhage (red arrow) and intraventricular hemorrhage (white arrow).

On motor exam, her left upper extremity showed some effort against gravity but hit the bed before 10 seconds. Motor exam of right upper extremity was normal. Motor exam of both lower extremities exhibited drift but did not hit the bed before five seconds. She otherwise had no sensory deficits, no ataxia, no aphasia, and no dysarthria. Furthermore, there was no extinction to double simultaneous stimuli testing. Her National Institutes of Health Stroke Scale was four. A computed tomography (CT) of her brain showed pneumocephaly, SDH, SAH, and intraventricular hemorrhage (Images 1 and 2).

The patient was admitted to the intensive care unit (ICU). Her blood pressure was managed with labetalol. A CT angiogram (CTA) of the head did not show any aneurysm. She was treated with nimodipine in the ICU. Four days after the procedure repeat brain CT showed resolution of pneumocephaly and intracranial hemorrhage. Prior to discharge her repeat neurologic exam showed resolution of all deficits.

## DISCUSSION

Dural puncture after CEI is a known complication.^2^ There have been case reports of intracranial complications after epidural injectons.^4^,^5^,^6^ However, literature review failed to find reports of SDH, SAH, and pneumocephaly in the same patient after CEI. This is a unique case report describing all three complications in the same patient. Pneumocephalus is thought to occur as a result of inadvertent injection of air into the subdural space.^4^ Furthermore, SDH has been described following lumbar puncture.^5^ It is postulated to result from direct dural puncture.^5^

Straining actions such as coughing may increase cerebrospinal fluid (CSF) pressure at puncture site leading to CSF leak from the site. The loss of CSF can lead to shift in brain tissue, which can result in shearing of blood vessels. The shearing of blood vessels can lead to their rupture, which can cause SDH. The patient had vomited post procedure. This may have caused CSF leak from an inadvertent dural puncture site triggering the chain of events leading to SDH. In addition, SAH has also been described following lumbar puncture.^6^,^7^ SAH is thought to result from unintentional direct puncture of a spinal vessel causing it to leak.^6^,^7^ The most common etiology of spontaneous SAH is an aneurysm rupture.^8^ The patient did have a CTA of the head, which did not show any aneurysms.

**CPC-EM Capsule**

What do we already know about this clinical entity?

*Cervical epidural injections are known to cause serious complications such as brain and spinal cord infarctions*.

What makes this presentation of disease reportable?

*We describe the complications of subdural hemorrhage, subarachnoid hemorrhage, and pneumocephaly in the same patient after a cervical epidural injection*.

What is the major learning point?

*Emergency physicians should consider intracranial complications in patients who present with a headache after cervical epidural injection*.

How might this improve emergency medicine practice?

*Knowing the potential complications after cervical epidural injections will allow for rapid initiation of diagnostic work-up*.

## CONCLUSION

It is important to consider intracranial complications, when a patient presents to ED with headache following a CEI. The differential diagnosis should include SDH, SAH, and pneumocephalus. Evaluation should include head CT. Treatment is supportive in the ED and likely admission to ICU.
